# Editorial: Assessment and diagnosis of bipolar disorder: Volume II

**DOI:** 10.3389/fpsyt.2022.1065442

**Published:** 2022-11-07

**Authors:** Andrea Fagiolini

**Affiliations:** Department of Molecular and Developmental Medicine, University of Siena School of Medicine, Siena, Italy

**Keywords:** bipolar, assessment, diagnosis, symptoms, neuroimaging

Bipolar disorder (BD) is often regarded as an acute condition, mainly characterized by the presence of episodes of hypomania and mania. However, it is now increasingly clear that this condition: (1) tends to be chronic, with most patients that spend a substantial proportion of time with depressive or manic symptoms; (2) is often resistant, or partially resistant, to treatment; and (3) is often burdened by subthreshold symptoms that persist between one acute episode and another.

Furthermore, there is growing evidence that the prevalence of bipolar disorder can go well beyond the 1–2% prevalence that is usually attributed to this disease. Reasons for this underestimation include difficulties in making an early diagnosis and difficulty in making a differential diagnosis from disease such as major depressive disorder, anxiety disorders, personality disorders, etc. In this collection of articles, some of the topics mentioned above are addressed, including:

1. The differences in the seasonal variations of symptoms, between patients with bipolar disorder and patients with major depressive disorder (Kong et al.);
2. Findings from a neuroimaging study on peculiar characteristics in some areas of the cerebral cortex of patients with bipolar disorder (Okamoto et al.);
3. The evaluation of the clinical characteristics of dissociative symptoms and of their impact on the clinical course of the disease, including. the response to treatment (Steardo et al.);
4. The differences between patients with bipolar disorder and patients with major depressive disorder in the brain white matter structure (Koreki et al.);
5. The clinical and neurobiological trajectories of children and adolescents with bipolar disorders and high-risk unaffected offspring (Paim Diaz et al.).

I am grateful to the Authors who contributed to this research topic and hope that the readers will appreciate and find their contribution useful for their clinical practice and research on this topic.

## Author contributions

The author confirms being the sole contributor of this work and has approved it for publication.

## Conflict of interest

The author declares that the research was conducted in the absence of any commercial or financial relationships that could be construed as a potential conflict of interest.

## Publisher's note

All claims expressed in this article are solely those of the authors and do not necessarily represent those of their affiliated organizations, or those of the publisher, the editors and the reviewers. Any product that may be evaluated in this article, or claim that may be made by its manufacturer, is not guaranteed or endorsed by the publisher.

